# Dynamics of Posttranslational Modifications of p53

**DOI:** 10.1155/2014/245610

**Published:** 2014-05-12

**Authors:** Qing-Duan Fan, Guang Wu, Zeng-Rong Liu

**Affiliations:** ^1^Institute of Systems Biology, Shanghai University, 99 Shangda Road, Shanghai 200444, China; ^2^College of Fundamental Studies, Shanghai University of Engineering Science, 333 Longteng Road, Shanghai 201620, China; ^3^Guangxi Academy of Sciences, 98 Daling Road, Nanning, Guangxi 530007, China; ^4^DreamSciTech, Apartment 207, Zhencaili 26, Zhujiang Road, Hexi District, Tianjin 300222, China

## Abstract

The latest experimental evidence indicates that acetylation of p53 at K164 (lysine 164) and K120 may induce directly cell apoptosis under severe DNA damage. However, previous cell apoptosis models only studied the effects of active and/or inactive p53, that is, phosphorylation/dephosphorylation of p53. In the present paper, based partly on Geva-Zatorsky et al. (2006) and Batchelor et al. (2008), we propose a new cell apoptosis network, in which p53 has three statuses, that is, unphosphorylated p53, phosphorylated p53, and acetylated p53. The time delay differential equations (DDEs) are formulated based on our network to investigate the dynamical insights of p53-induced cell apoptosis. In agreement with experiments (Loewer et al. (2010)), our simulations indicate that acetylated p53 accumulates gradually and then induces the proapoptotic protein Bax under enough DNA damage. Moreover, phosphorylated p53 oscillates and initiates cell repair during DNA damage.

## 1. Introduction 

The tumor suppressor p53, a multifunctional transcription factor, plays an essential role in regulating cellular processes including cell cycle arrest and apoptosis [1]. The importance of p53 also lies in its mutation in over 50% of human cancers. There are three main routes, namely, DNA double-strand break (DSB), ultraviolet light (UV), and oncogenes, which can result in an increase in p53 expression. Experiments demonstrate that cell outcomes depend on the extent of DNA damage, which probably decides the number of p53 pulses or p53 oscillation [2, 3]. Experimentally, p53 regulates cell cycle protein p21, PUMA, BCL-2, PTEN, Bax, Bak, and so on [4], and a cell survives when DNA damage is reparable or commits suicide when irreparable. That is to say, a cell has these two means of avoiding cancer under DNA damage [5]. It follows that the mechanism of repair or apoptosis is closely linked with p53.

There are large quantities of experimental and theoretical researches on p53 networks, in which hundreds of genes and their corresponding proteins are involved. Consequently, the networks include many positive and negative feedback loops acting upon p53. The most prominent of them is the feedback loop between p53 and Mdm2 (mouse double minute 2), which has been considered in many dynamical models. p53 and Mdm2 show nondecaying oscillations in an individual cell, while demonstrating decaying oscillations in cell population, which may be ascribed to aggregate behavior of cells [1]. The ATM- (Ataxia Telan-giesctasia Mutated-) p53-wip1 (wild-type p53-induced phosphatase 1) feedback loop plays an important role in the generation of p53 pulses [6]. These intricate positive and negative feedback loops display various dynamical behaviors [7]. The reliable and flexible mechanism can avoid the premature apoptosis resulting from fluctuations in p53 levels. It is indicated that p53 is modified in a progressive manner and that p53 is divided into p53-arrest and p53-apoptosis in the integrative model [8]. Moreover, high constant levels of active p53 may trigger apoptosis quickly once the decision favoring death is made in seriously damaged cells.

There are several “protein-protein” and “protein-mRNA” dynamical models which describe in detail intracellular signalling of the protein p53. Many theoretical results are obtained in determinate systems. Based on two compartments, nucleus and cytoplasm, an ODEs model is formulated to exhibit that the accumulation of p53 after triggering of ATM under DNA damage. The model also shows robustness of the protein oscillatory dynamics in response to different cellular environments [9]. A set of ordinary differential equations in single cell level shows p53 oscillations in each compartment, nucleus or cytoplasm, and between the two compartments [10]. Based on a sequence of precisely timed drug additions, the authors formulate a computational model, which shows that the dynamic of p53 changes from a pulse to a sustained response [11]. There are also quite a few stochastic systems, where the stochasticity of regulation on p53 shows high heterogeneity and stochastic character of single-cell response [12–15]. Up to now, only phosphorylation modification has been considered in dynamical models.

In fact, a cell is regulated accurately by many posttranslational modifications of p53, which can initiate a program of cell repair or apoptosis at different levels of DNA damage. Methylation of p53 facilitates its subsequent acetylation and protects p53 from ubiquitination [16]. Phosphorylation of p53 is important for inducing p21, a prime inhibitor of cell cycle. Recently, experiments find that a number of external and internal insults induce acetylation and accumulation of p53, via MYBBP1A, RPL5, and RPL11, without phosphorylation [17–19]. It is shown that acetylation of p53 at K164 and K120 may promote cell apoptosis rather than cell arrest [20, 21]. It is observed that p53 may fundamentally switch from pulsing under slight damage to monotonic increase under severe damage [22]. However, there is no corresponding theoretical result about the dynamics of acetylation of p53 up to now.

In the paper, we distinguish functionally the effect of acetylation from phosphorylation of p53 and develop the DDEs of p53 transcriptional regulatory networks based on new experiments [18, 22] and related researches [1, 2, 6, 8, 12]. We pay special attention to the effect of acetylation of p53 and the proapoptotic protein Bax in the case of DNA damage. In agreement with experiments, our simulations indicate that acetylated p53 accumulates gradually with serious DNA damage and induces Bax when p53 surpasses a level.

## 2. Methods and Models 

**Figure 1:**
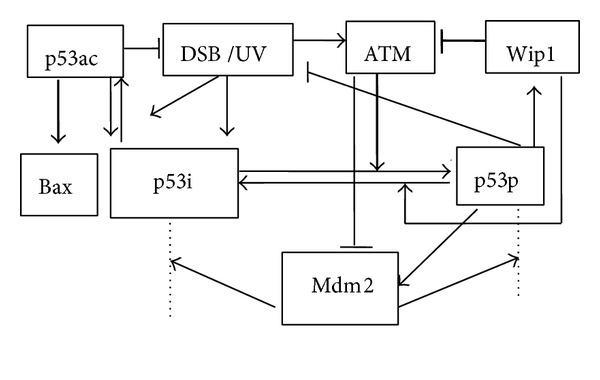
Schematic depiction of the model for the p53 networks in response to DNA damage. Transcription regulation is denoted in thick line. Degradation is denoted in dotted line. Sharp and blunt arrow denote activation and suppression, respectively. ATM's catalyzing phosphorylation of Mdm2, allowing Mdm2 easier to degrade, is regarded as repressive.

Methylation allows p53 to be inactive, in normal circumstances p53 is samilarly inactive, so we may regard the initial status of p53 to be inactive without consideration of methylation. Dynamical models of two statuses of p53, that is, inactive p53 and active p53/phosphorylated p53, have been studied extensively [2, 3, 6, 8]. It is indicated that active forms of p53, such as phosphorylated p53 and acetylated p53, have different dynamics and functions experimentally [18]. Interestingly, p53 can be acetylated and accumulates without phosphorylation [17]. Acetylation of p53 on K120 is crucial to p53 dependent apoptosis but is dispensable for p53-mediated growth arrest [19]. In another experiment, acetylation-defective of p53 at k120 can selectively block the transcription of proapoptotic target genes such as Bax and PUMA but has no obvious effect on cell cycle inhibitor p21 [16]. Accordingly, it is necessary that we explore the relation between the means of modifications and cell outcomes. It is shown that p53 may transcribe a few proapoptotic genes such as PUMA, Noxa, Bax, and Bid under excessive DNA damage. Moreover, Bax is very much related to cell apoptotic [23]. For simplicity, we choose Bax as a proapoptotic marker. Because both cell repair and cell apoptosis can lessen DSB; to put it in another way, p53ac and p53p can be regarded as inhibiting DSB. Based on preexisting researches [2, 3, 6], we obtain the following modular schematic depiction in Figure 1.

Considering the tetramer of p53 as a transcription factor, p53-induced Bax is characterized by a Hill function. When phosphorylation and dephosphorylation of p53 are considered separately, a near-optimal switch is possible via Hill equation, where the Hill coefficient equals the number of phosphorylation sites [24]. It is shown that p53 can be phosphorylated at sites S15, T18, S20, S37, S378, and S392 [25]; but Hill coefficient usually ranges between 2 and 4 [24]. Here we denote the ATM-induced phosphorylation of p53 and wip1-induced dephosphorylation of p53 via Hill equation. For simplicity, Hill coefficients take 4. Specially, wip1-induced dephosphorylated of ATM is denoted by Hill equation, where Hill coefficient takes 2 for ATM protein is a dimer. Active p53 has a weaker interaction with Mdm2 than inactive p53 and hence a lower degradation rate [1].

Phosphorylated p53 has a lower degradation rate than inactive p53 because the binding of it with Mdm2 is weaker than inactive p53 [1]. Additionally, the acetylated residues cannot be ubiquitylated by Mdm2 [26]. In the model, two delays are considered because the transcription of Mdm2 by p53 needs time [2] and wip1 expression with a delay would allow p53-induced cell repair [1]. Sustained damage leads to acetylation and accumulation of p53 [17]; however, no acetylation was detected in response to a transient and low-level damage (Figure 6(d) in [18]). In other words, p53 is acetylated only when DNA damage surpasses a certain level, *θ*_0_. It is shown that the transcription rate of p53 is independent of DNA damage; moreover, there exists an increase in translation rate of p53 following gamma irradiation, so signal strength, ATM, can be denoted by *θ*(*x*). Here *θ*(damage) equals 1 if damage exists, zero otherwise [2]. According to Figure 1 and the dynamical model in [2, 3, 6], we further formulate a set of DDEs:

(1) dp53idt=bp−ampiMdm2·p53i−bspATMp53in1tsn+p53in1 −apip53i+awpawip1p53pn1tin1+p53pn1 −bpacp53i max⁡(DSB−θ0,0)+adepacp53ac,

(2) dp53pdt=bspATMp53in1tsn1+P53in1−ampaMdm2·p53p −awpawip1p53pn1tin1+p53pn1,

(3) dMdm2dt=bmi+bmp53p(t−τ1)−asmATM·Mdm2 −amMdm2,

(4) $$\begin{array}{c} \frac{d\text{wip}1}{dt}=b_{i}\text{p}53\text{p}\left(t-\tau_{2}\right)-a_{i}\text{wip}1 \end{array}$$

(5) $$\begin{array}{c} \frac{d\text{ATM}}{dt}=b_{s}\theta \left(\text{DSB}\right)-a_{\text{is}}\text{wip}1\frac{\text{AT}{\text{M}}^{n2}}{ti^{n2}+\text{AT}{\text{M}}^{n2}}-a_{s}\text{ATM}, \end{array}$$

(6) dp53acdt=bpacp53i max⁡(DSB−θ0,0) −apacp53ac−adepacp53ac,

(7) $$\begin{array}{c} \frac{d\text{Bax}}{dt}=b_{\text{Bax}}\frac{\text{p}53\text{a}{\text{c}}^{n1}}{ta^{n1}+\text{p}53\text{a}{\text{c}}^{n1}}-a_{\text{Bax}}\text{Bax}, \end{array}$$

(8) $$\begin{array}{c} \frac{d\text{DSB}}{dt}=-k_{\text{rep}}\text{DSB}\left(\text{p}53\text{p}+\text{p}53\text{ac}\right), \end{array}$$

where p53i, p53p, and p53ac represent inactive p53, phosphorylated p53, and acetylated p53, respectively. In (1), the first term represents p53i synthesis; the second one, catalytic degradation of Mdm2; and the third one, phosphorylation; the fourth to the last one describe self-degradation, dephos- phorylation, and acetylation, respectively. In (2), the first term denotes phosphorylation of p53 by ATM; the second one, ubiquitination by Mdm2; and the third one, wip-dependent dephosphorylation. In (3), the first term represents Mdm2 synthesis speed; the second one, activating Mdm2 via p53p, where the delay, *τ*_1_, denotes the time for the transport of p53 from cytoplasm to nucleus and the transcription of Mdm2; the third one, catalyzation of ATM; and the last one, self-degradation. In (4), the first term represents activating wip1 via p53, where the delay, *τ*_2_, is introduced owing to the time for the transport of p53 and the transcription of wip1 by p53 and the second one, self-degradation. In (5), the first term represents exciting ATM induced by DSB; the second one, dephosphorylation by wip1; and the third one, self-degradation. In (6), the first term represents p53 acetylation caused by DSB, wherein max⁡(*x*) refers to the maximum function and the second one, self-degradation of p53ac. In (7), the first term represents Bax induced by p53 and the second one, self-degradation of Bax. (8) denotes the reduction rate of DSB owning to cell repair and apoptosis. For simplicity, all the parameters in the system consisting of (1)–(8) are listed in Table 1.

## 3. Results 

Various exogenous or endogenous stimuli can generate damaged DNA. DSBs and UV are the main types of stimuli, which can activate p53 and subsequently command cell outcomes. DSBs are discussed in two cases, that is, pulsing and repairable DSBs, so we simulate the model according to three cases of DNA damage.

**Figure 2:**
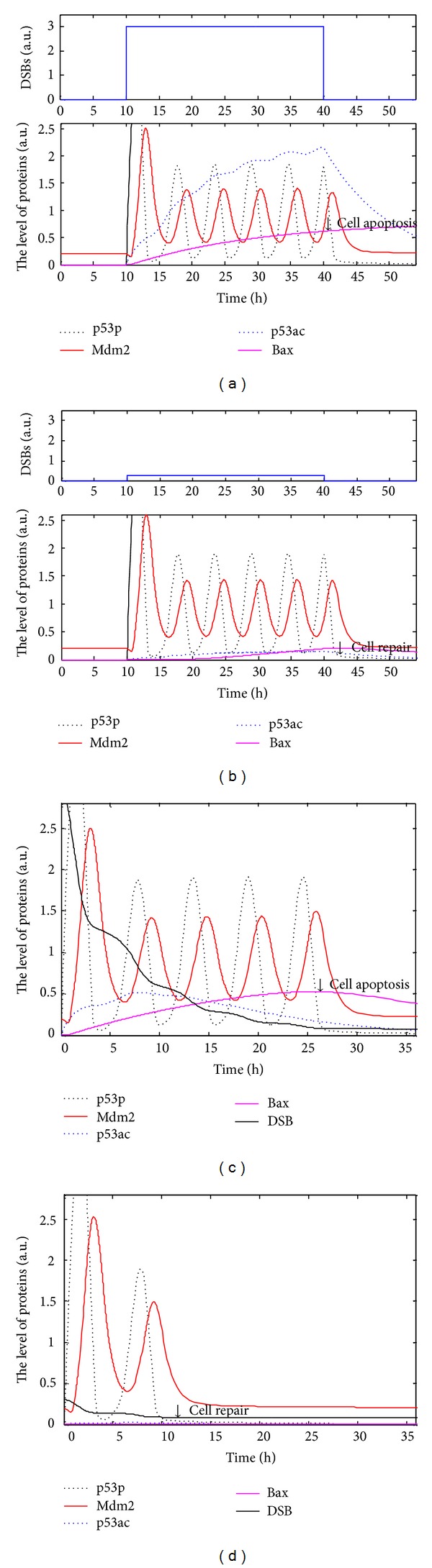
Simulations of the p53 regulatory networks under different types of DSBs. Black dotted lines indicates concentration of p53p; red solid lines Mdm2; blue dotted lines p53ac; carmine solid lines Bax; and black solid line DSB. (a, b) Pulsating DSB. (a) Pulse of DSBs takes 3 (a) and 0.3 (b). (c, d) DNA repair via p53 is considered. The initial DSBs take 3 (c) and 0.3 (d). (a, c) p53p oscillates while p53ac accumulates and induces cell apoptosis. (b, d) p53p oscillates while p53ac is hardly expressed during cell repair.

Firstly, we consider the system equations ((1)–(7)) with DSBs at pulsing level. When 10 < *t* < 40, DSBs take 3, otherwise DSBs take 0. *θ*_0_, the threshold of DSB for acetylating p53, takes arbitrarily 0.1. The initial conditions of p53i and Mdm2 take 1 and 0.2, respectively; the others take 0. The numerical simulations with Matlab 7.10 (Mathworks) are shown in Figure 2(a), which indicates that p53p has several oscillations with a constant period of about 6 hs and that p53ac accumulates. When p53ac surpasses a level, 0.2 or so, it activates proapoptosis protein Bax. From a biological standpoint, it is reasonable that Bax is activated when p53ac reaches a sufficient level. It is better that the threshold can be testified by experiments. In our simulations, Bax is up to 0.6, which is a rather high level, and we think that cell apoptosis should occur. Experimentally, DSBs are basal in proliferating cell, but cell apoptosis does not occur. p53ac hardly expresses when DSBs are smaller than 0.3, and Bax is also the case (simulations are shown in Figure 2(b)).

Secondly, DSBs decrease under cell pair or cell apoptosis. We consider the system equations (1)–(8) with the parameters and initial conditions set in Table 1 and get numerical simulation shown in Figures 2(c) and 2(d). It is shown that p53p oscillates owing to decreasing DSBs, with a constant period similar to that in the system equations (1)–(7), while p53ac accumulates under serious DNA damage. p53ac ascends and then descends with the decrease of DSBs. When p53ac surpasses a certain level, 0.2 or so, it induces Bax. These simulation results, such as a sufficient level of p53ac activating Bax, are consistent with the biological fact that activation of some proteins need enough inducer. With parameters set in Table 1, simulations show that in the system p53p probably has 5 pulses when [DSBs]_0_ is 3 (Figure 2(c)). When [DSBs]_0_ drops to 0.3, p53ac and Bax are hardly expressed, and p53p has 2 pulses (Figure 2(d)). In the cases that [DSBs]_0_ is 1 or 20, cell has only 4 pulses, but cells have different outcomes, that is, cell repair and cell apoptosis (simulations not shown). The smaller [DSBs]_0_ is, the smaller p53ac and the number of pulse of p53p are. On the contrary, the greater [DSBs]_0_ is, the faster p53ac accumulates but the smaller the number of pulse of p53p is, which appear to show that cell apoptosis is faster.

**Figure 3:**
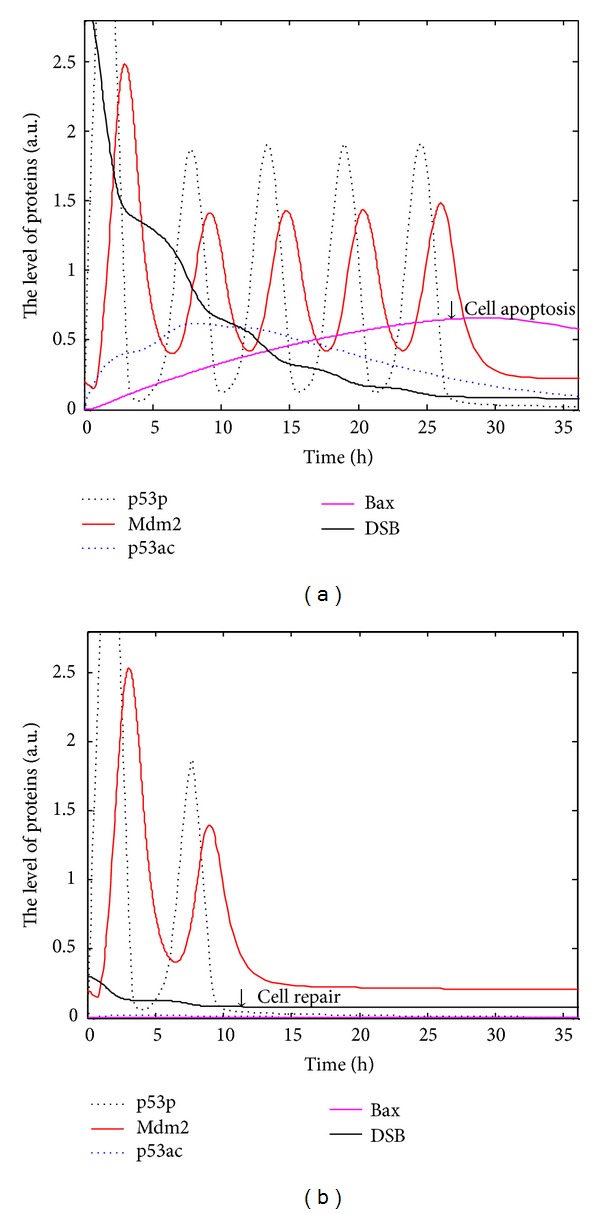
Parameters robustness of the p53 regulatory networks. Black dotted line indicates concentration of p53p; red solid line Mdm2; blue dotted line p53ac; carmine solid line Bax; and black solid line DSB. (a) Production rates decrease 10% and degradation rates increase 10% in Figure 2(c). (b) Production rates increase 10% and degradation rates decrease 10% in Figure 2(d).

Now we consider the robustness of the system parameters. Simulations of the system shown in Figures 2(c) and 2(d) with new parameters perturbation and with cited parameters are shown in Figure 3. When *b*_pac_,  *b*_Bax_, and *a*_depac_ increase by 10%, and *a*_pac_ and *a*_Bax_ decrease by 10% in parameters of Figure 2(c), simulations of the p53 networks are shown in Figure 3(a). When *b*_pac_,  *b*_Bax_, and *a*_depac_ decrease by 10% and *a*_pac_,  *a*_Bax_, and *k*_rep_ increase by 10% in parameters of Figure 2(d), simulations are shown in Figure 3(b). Our new parameters increase or decrease by 10%, qualitative characteristics change little except for the position of the equilibrium point (Figure not shown). It is evident that steady points of p53i and Mdm2 do not change much comparing to that of using the initial parameters values. However, p53p and Mdm2 still oscillate while p53ac and Bax accumulate.

At last, when DNA is damaged by ultraviolet rays (UV), ATR, instead of ATM, phosphorylates p53 and Mdm2, except that dephosphorylation of wip1 on ATR is dispensable, the other pathways do not change. Consequently, we have the following differential equations in response to UV:

(9) dp53idt=bp−ampiMdm2 p53i−bspATRp53in1tsn1+p53in1 −apip53i+awpawip1p53pn1tin1+p53pn1 −bpacp53i  θ(UV−θ0),

(10) dp53pdt=bspATRp53in1tsn1+p53in1−ampaMdm2 ·p53p−awpawip1p53pn1tin1+p53pn1,

(11) dMdm2dt=bmp53p(t−τ1)+bmi −asmATR Mdm2−amMdm2,

(12) $$\begin{array}{c} \frac{d\text{ATR}}{dt}=b_{s}\text{UV}-a_{s}\text{ATR}, \end{array}$$

(13) dp53acdt=bpacp53i  θ(UV−θ0) −apacp53ac−adepacp53ac,

where (9)–(13) are based on [6].

**Figure 4:**
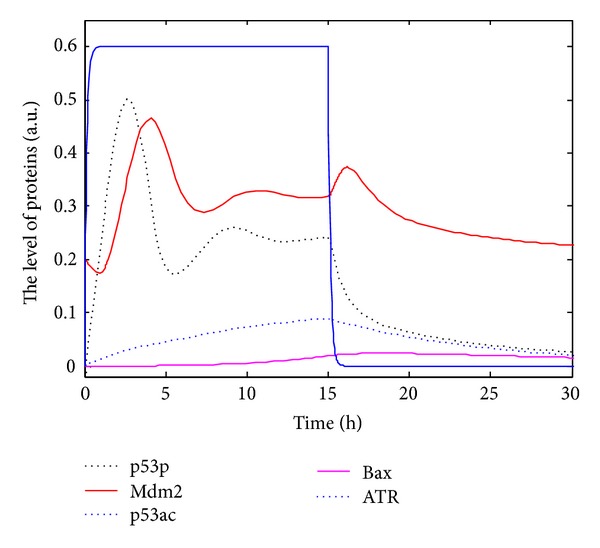
The dynamics of all kinds of p53 networks in response to UV = 8. Black dotted line indicates concentration of p53p; red solid line Mdm2; blue dotted line p53ac; carmine solid line Bax; and blue solid line ATR.

We regard damage capability of UV as one-tenth of DSB. Paramters *b*_pac_, *a*_pac_, *b*_Bax_, *a*_Bax_, *θ*_0_ and the initial conditions are set and shown in Table 1. Take UV = 8 for 0 ≤ *t* < 15, and take 0 for *t* ≥ 15 as [6], the simulations of (4), (7), and (9)–(13) are shown in Figure 4. It is indicated that during slight DNA damage, p53p exhibits a small pulse so as to induce cell cycle arrest/cell repair, and then returns to the initial condition. It is worth noting that p53ac and cell apoptosis are little when UV ≤ 10, which is consistent with experimental results [6]. Total p53 has a pulse as phosphorylated p53 does and then returns to the basal level.

## 4. Conclusion 

The tumor suppressor p53, a most frequently mutated protein in cancer cells, is a key regulator in cell cycle. The latest experiments [17, 18, 24] show that posttranslational modifications of p53, such as phosphorylation and acetylation, are closely linked with cell repair and cell apoptosis. In order to interpret the experimental phenomenon, we develop the regulatory networks and the DDEs model and discuss the dynamics of modifications of p53. Experimentally, acetylation of p53 at K120 and K164 plays an important role in regulating proapoptotic protein [16–19]. It is indicated exactly that p90 is critical to p53-mediated cell apoptosis through promoting acetylation of p53; moreover, p90 has no obvious effects on p53-mediated cell cycle arrest but it is specifically needed for p53-mediated apoptosis [27]. The phenomena that p53ac accumulates and activates proapoptotic protein Bax only under serious DNA damage (Figures 2(a) and 2(c)) and that the pulses of p53p and a little p53ac allow cell to reenter cell cycle under slight DNA damage (Figures 2(b) and 2(d)) are consistent with the latest experiments [2, 6, 18]. The robustness analysis of the model (Figure 3) shows that the accumulation of p53ac and the number of pulse of p53p depend on the extent of DNA damage. The number of pulses of p53p, which means cell repair, should lie on cell repair or cell apoptosis under different levels of DNA damage [28]. UV can lead to single strand break, a kind of slight DNA damage, which usually activates p53p and allows cell to reenter cell cycle (Figure 4). Accordingly, posttranslational modifications of p53 can help us know when and why a cell selects programmed cell death or cell repair. In other words, posttranslational modifications of p53 will be beneficial to the treatment of tumors as an innovative therapeutic strategy.

Since cell kinetics is complicated and precisely accurate, it is very difficult to consider all details of posttranslational modifications of p53 [25, 27]. Reference [29] shows phosphorylation may allow p53 stabilization, enhancement of DNA-binding, and activation of its cell-cycle arrest pathway, while acetylation may allow p53 to activate its apoptotic pathway. Yet there are exceptions. For example, phosphorylation at S46 is critical to the induction of proapoptotic genes p53AIP1 (p53-regulated Apoptosis-Inducing Protein 1), but it is not required for the induction of cell cycle inhibitor p21. Phosphorylation and acetylation of p53 probably have synergistic effects on cell cycle, such as acetylation of p53 at k320 may also induce p21 and repress apoptosis [30]. Methylation may allow p53 to be a transcriptionally inactive state; however, methylation of p53 facilitates its subsequent acetylation and protects p53 from ubiquitination [16]. Neddylation and sumoylation have not been demonstrated to affect p53 stability yet. Additionally, p53 may regulate the transcription expression of many miRNAs, such as MiR-34 and MiR-200. On the other hand, the expression and modification of p53 are also regulated by quantities of MiRNAs [31, 32]. Accordingly, the mechanism of posttranslational modification needs exploration for a long time both in experiment and in theory, such as the mechanism of randomness of protein expression. Our further work will focus on more accurate posttranslational modifications of p53 in cell cycle.

## Figures and Tables

**Table 1:** The meaning and values of parameters in the systems (1)–(8).

Parameter	Biological meaning	Value
*b*_*p*_	Inactive p53 production rate	3
*b*_sp_	p53 saturating phosphorylate rate by ATM/ATR	10*/1
*b*_*m*_	p53-dependent Mdm2 production rate	0.9
*b*_mi_	p53-independent Mdm2 production rate	0.2*
*b*_*i*_	Inhibitor Wip1 production rate	0.25*
*b*_*s*_	Signal ATM production rate	10*
*b*_pac_	Acetylation speed of p53 under DSB/UV damage	0.1/0.02
*b*_Bax_	Saturating production rate of Bax	0.04
*a*_mpi_	Mdm2-dependent p53 inactive degradation rate	5*
*a*_pi_	Inactive p53 degradation rate	2*
*a*_mpa_	Mdm2-dependent active p53 degradation rate	0.35
*a*_sm_	Signal-dependent Mdm2 inactivation rate	0.5
*a*_wpa_	Wip1-dependent dephosphorylation rate of p53	2.8
*a*_*m*_	Mdm2 degradation rate	1*
*a*_*i*_	Inhibitor Wip1 degradation rate	0.7*
*a*_*is*_	Wip1-dependent Signal degradation rate	50*
*a*_*s*_	ATM degradation rate	7.5*
*a*_pac_	acetylation of p53 degradation rate	0.05
*a*_depac_	deacetylation rate of p53	0.05
*a*_Bax_	Bax degradation rate	0.04
τ_1_	Time delay of Mdm2 transcription by p53	0.7*
τ_2_	Time delay of Wip1 transcription by p53	1.25*
*ta*	Concentration of Bax for half maximal p53ac production	0.15
*ts*	Concentration of ATM for half-maximal p53 production	1*
*ti*	Concentration of wip1 for half-maximal Signal degradation	0.2*
*θ*_0_	The threshold of DSBs for acetylating p53	0.1
*k*_rep_	Repair rate of DSBs	0.1
*n*_1_	Hill coefficients for phosphorylation and dephosphorylate of p53	4
*n*_2_	Hill coefficients for dephosphorylation of ATM by wip	2
[p53i]_0_	The initial condition of inactive p53	1
[p53p]_0_	The initial condition of phosphorylated p53	0
[Mdm2]_0_	The initial condition of Mdm2	0.2
[wip1]_0_	The initial condition of wip1	0
[ATM]_0_	The initial condition of ATM	0
[p53ac]_0_	The initial condition of acetylated p53	0
[Bax]_0_	The initial condition of Bax	0
[DSBs]_0_	The initial conditions of two type of DSBs	3/0.3**

*denotes parameters from [2, 6], the others are estimated. **denotes serious/slight DSBs.
